# Reference Cluster Normalization Improves Detection of Frontotemporal Lobar Degeneration by Means of FDG-PET

**DOI:** 10.1371/journal.pone.0055415

**Published:** 2013-02-25

**Authors:** Juergen Dukart, Robert Perneczky, Stefan Förster, Henryk Barthel, Janine Diehl-Schmid, Bogdan Draganski, Hellmuth Obrig, Emiliano Santarnecchi, Alexander Drzezga, Andreas Fellgiebel, Richard Frackowiak, Alexander Kurz, Karsten Müller, Osama Sabri, Matthias L. Schroeter, Igor Yakushev

**Affiliations:** 1 LREN, Département des Neurosciences Cliniques, CHUV, Université de Lausanne, Lausanne, Switzerland; 2 Clinic of Cognitive Neurology, University of Leipzig, Leipzig, Germany; 3 Max-Planck-Institute for Human Cognitive and Brain Sciences, Leipzig, Germany; 4 LIFE – Leipzig Research Center for Civilization Diseases, University of Leipzig, Germany; 5 Mind Brain Institute, Charité and Humboldt University, Berlin, Germany; 6 Consortium for Frontotemporal Lobar Degeneration, Germany; 7 Department of Nuclear Medicine, University of Leipzig, 0Leipzig, Germany; 8 Department of Psychiatry and Psychotherapy, Technische Universität München, Munich, Germany; 9 Department of Nuclear Medicine, Technische Universität München, Munich, Germany; 10 Department of Neurological, Neurosurgical and Behavioral Sciences, University of Siena, Italy; 11 University Medical Center Mainz, Mainz, Germany; 12 Neuroepidemiology and Ageing Research Unit, School of Public Health, Faculty of Medicine, The Imperial College of Science, Technology and Medicine, London, United Kingdom; Federal University of Rio de Janeiro, Brazil

## Abstract

Positron emission tomography with [18F] fluorodeoxyglucose (FDG-PET) plays a well-established role in assisting early detection of frontotemporal lobar degeneration (FTLD). Here, we examined the impact of intensity normalization to different reference areas on accuracy of FDG-PET to discriminate between patients with mild FTLD and healthy elderly subjects. FDG-PET was conducted at two centers using different acquisition protocols: 41 FTLD patients and 42 controls were studied at center 1, 11 FTLD patients and 13 controls were studied at center 2. All PET images were intensity normalized to the cerebellum, primary sensorimotor cortex (SMC), cerebral global mean (CGM), and a reference cluster with most preserved FDG uptake in the aforementioned patients group of center 1. Metabolic deficits in the patient group at center 1 appeared 1.5, 3.6, and 4.6 times greater in spatial extent, when tracer uptake was normalized to the reference cluster rather than to the cerebellum, SMC, and CGM, respectively. Logistic regression analyses based on normalized values from FTLD-typical regions showed that at center 1, cerebellar, SMC, CGM, and cluster normalizations differentiated patients from controls with accuracies of 86%, 76%, 75% and 90%, respectively. A similar order of effects was found at center 2. Cluster normalization leads to a significant increase of statistical power in detecting early FTLD-associated metabolic deficits. The established FTLD-specific cluster can be used to improve detection of FTLD on a single case basis at independent centers – a decisive step towards early diagnosis and prediction of FTLD syndromes enabling specific therapies in the future.

## Introduction

In brain imaging by means of positron emission tomography (PET) and single-photon emission computed tomography (SPECT), scaling of tracer uptake to a reference region is in most cases essential for analyses of non-quantitative data. An ideal reference region should not be affected by brain pathology and should be easy to image/analyse. The choice of the appropriate reference region is especially problematic in subjects with neurodegenerative disorders who show early metabolic and perfusion deficits [1]–[5].

Using statistical parametric mapping (SPM), we have recently proposed a data-driven method for normalization of [18F] fluorodeoxyglucose (FDG) uptake in cases with preclinical and manifest Alzheimer's disease (AD) dementia [6]. As compared with traditional intensity normalization to an *a priori* defined reference region, the reference cluster (RC) is defined by a contrast showing areas with increased activity in patients relative to controls after global mean normalization. This RC approach proved to detect AD-related hypometabolism in a more sensitive manner than traditional ROI-based normalizations [6]. Originally developed on clinical image data, the method was soon validated in simulation studies and extended to perfusion studies in Parkinson's disease (PD), another common neurodegenerative disorder [7]–[9].

To be applicable in clinical settings, any diagnostic test must be robust to variability in clinical presentation/assessments and methodological factors. To this end, we performed a bi-central study with a cross-validation design in frontotemporal lobar degeneration (FTLD). As compared to AD and PD, FTLD is characterized by a substantially higher heterogeneity in respect to histopathological, clinical, and imaging presentation [10]–[12]. FDG-PET plays a well-established role in assisting early detection and differentiation of this severe neurodegenerative disorder [13]–[25], after AD the second most common cause of presenile dementia [26]. Furthermore, as compared to previous single-center applications of the RC normalization in AD and PD [6], [27], [28], here we assess performance of the method in cross-center settings. I.e., a RC obtained at one center is applied for data normalization from another center. Results of this data-driven approach are then compared with normalization to common reference regions.

In this work, we examine the impact of intensity normalization to cerebellum (CBL), primary sensorimotor cortex (SMC), cerebral global mean (CGM) and to RC on the accuracy of FDG-PET to detect FTLD-specific metabolic deficits and to discriminate between patients with mild FTLD and healthy subjects.

## Methods

### Subjects

Patients were retrospectively identified from a database of subjects from the memory clinic of the Department of Psychiatry at the Technische Universität München (hereafter referred to as center 1) and from the Clinic of Cognitive Neurology at the University of Leipzig (thereafter referred to as center 2). In both centers all patients were diagnosed according to Neary diagnostic criteria of FTLD [29]. The diagnosis was based on the information from a thorough neurological and psychiatric examination, informant interview, routine blood sampling, MRI, and FDG-PET imaging. Only patients with a Mini-Mental-State Examination (MMSE) score >21 were included. For two patients at center 2 MMSE was not available. In this case, a clinical dementia rating (CDR) score of less or equal to 1 was applied as an inclusion criterion [30]. Thus, we aimed to include patients with a mild disease severity only. Exclusion criteria were evidence for lesions due to stroke, traumatic head injury, brain tumor or inflammatory diseases on structural MRI. Using these criteria, 41 patients were included at center 1 and 11 at center 2.

The control group at center 1 (n = 42) consisted of elderly individuals without relevant psychiatric or neurological symptoms; subjects were only included if they did not report subjective memory complaints and if they did not show evidence for cognitive impairment.

The control group at center 2 (n = 13) included subjects who visited the Clinic of Cognitive Neurology at the University of Leipzig with subjective cognitive complaints, which were not objectively confirmed by a comprehensive neuropsychological and clinical evaluation.

This study was carried out in accordance with the latest version of the Declaration of Helsinki after the consent procedures had been approved by the local ethics committees of the University of Leipzig and of the medical faculty at the Technische Universität München. Written informed consent was obtained from all control subjects, all patients at center 2 and from most patients at center 1. Some patients at center 1 only gave oral informed consent for participation in the study as PET examination in patients was part of routine diagnostic work-up. Such a study-specific written consent was not obtained because all the procedures are included in a diagnostic work-up for suspected neurodegenerative disorder. Irrespective of this, all patients or their proxy gave a written consent for the PET examination. Additionally the process was documented by standard hospital documentation including an indication for the diagnostic work-up (due to suspected neurodegenerative disorder). The standard written (in German) consent includes the following items: 1) Aim of the study/PET examination, 2) Contraindications to the PET examination, 3) Procedure of the PET examination, 4) Expences and allowance, 5) Data protection, 6) Personal benefits, 7) Risks, complications and side-effects, 8) Insurance, 9) Responsible persons and consent to participate. As patients were studied with PET as a part of diagnostic work-up, items 4,6,8 and were not applicable. We confirm that all potential participants who declined to participate or otherwise did not participate were eligible for treatment and were not disadvantaged in any other way by not participating in the study. Demographic characteristics of participants are presented in Table 1.

**Table 1:** Subject group characteristics.

	Center 1		Center 2	
	Controls	FTLD	Controls	FTLD
Number	42	41	13	11
Male/Female	18/23	30/11	7/6	7/4
Age (years)	61.7±10.3	64.0±9.4	53.9±5.8	61.6±5.5
CDR (score)	-	-	0.2±0.2	0.7±0.2
MMSE (score)	-	25.5±1.8	-	26.0±2.1

Mean ± standard deviation. CDR Clinical Dementia Rating Scale, FTLD frontotemporal lobar degeneration, MMSE Mini Mental State Examination.

### Data acquisition

Scans were acquired under standard resting conditions with eyes closed at center 1 and in dimmed ambient light with eyes open at center 2, using a Siemens ECAT EXACT HR+ PET scanner (CTI, Knoxville, TN, USA).

At center 1, the acquisitions were performed in 3D mode with a total axial field of view of 15.52 cm and no interplane dead space. A sequence of three frames with a duration of 10, 5 and 5 min was acquired for each subject starting 30 min post injection. At center 2, data were acquired in 2D mode. A sequence of three frames (10 min each) was acquired for each subject starting 30 min post injection. Sixty-three slices were collected at both centers with an axial resolution of 5 mm full width at half maximum (FWHM) and in-plane resolution of 4.6 mm. After correction for attenuation, scatter, decay and scanner-specific dead time at both centers, images were reconstructed by filtered back-projection using a Hann-filter (center 1: cutoff frequency 0.5 cycles/projection element; center 2: 4.9 mm FWHM). Slices obtained had a resolution of 128×128 voxels with an edge length of 2.425 mm at center 1 and 2.45 mm at center 2.

### Data pre-processing

Statistical Parametric Mapping software (SPM8). http://www.fil.ion.ucl.ac.uk/spm/) implemented in Matlab 7.11 (MathWorks Inc., Sherborn, MA) was used for image processing and statistical analyses. After spatial realignment a mean image of the three frames was calculated for each subject. The image sets were spatially normalized using an in-house tracer-, scanner- and age-specific brain PET template (in MNI space) based on all available images, separately in each center. Normalized images with a voxel size of 2×2×2 mm were than smoothed with a Gaussian kernel of 12 mm FWHM. Regional value extraction for subsequent intensity normalization to CBL and to SMC was performed the automatic anatomical labeling atlas (AAL, [31]). All AAL regions belonging to cerebellar hemispheres and SMC were used as a single volume-of-interest for intensity normalization to CBL and SMC, respectively. The value of CGM was calculated using the global calculation function with default settings, as implemented in SPM [32], [33].

### Voxel-based analyses

**Figure 1:**
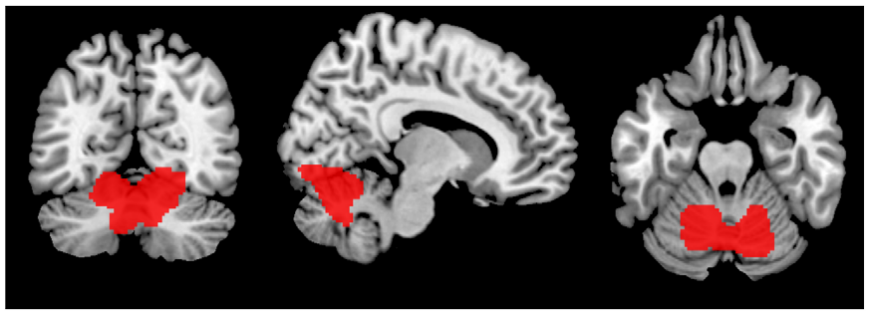
Region showing an increased glucose metabolism in frontotemporal lobar degeneration compared to control subjects in center 1 after normalization to cerebral global mean. A significance threshold of p<0.000001 family-wise error corrected at voxel level was applied. This region was used as reference cluster for the subsequent intensity normalization.

To obtain a representative FTLD-specific RC, we used data from center 1, as substantially more data sets were available. By analogy with the original work [6], we computed a contrast representing relative increases in the whole patient group (n = 41) compared to the control sample (n = 42), using normalization to CGM. As we had no experience with such a contrast in FTLD, no a priori probability threshold was chosen. Instead, we started with a threshold of p<0.05 FWE-corrected at voxel level and increased it until most of the resultant cluster volume could be clearly assigned to one specific anatomical region. Under the threshold of p<.000001 FWE-corrected at voxel level we obtained a single cluster that projected to the cerebellum. Of note, its volume was still sufficiently large, covering 4321 voxels. The cluster encompassed the medial and anterior parts of the cerebellar hemispheres as well as the cerebellar vermis (Figure 1). A small part of the cluster volume included the lower parts of the occipital cortex.

Further, we specified conventional contrasts representing relative decreases in the patient group (n = 41) relative to the control sample (n = 42). To evaluate the impact of normalization onto detection of FTLD specific hypometabolism, image intensity in these analyses was normalized to CGM, CBL, SMC, and RC as obtained above. To examine performance of normalization to RC in an independent sample, the same analyses were performed for data from center 2. Age and gender were included as nuisance variables in all analyses. A statistical threshold of p<.001 uncorrected at voxel level and p<0.05 FWE-corrected at cluster level was applied in all analyses. Extent threshold was set at k>100 contiguous voxels. Detected glucose hypometabolism was compared between the normalization procedures in terms of cluster size and maximum t-value. Anatomical labeling of significant clusters was performed using AAL [31] implemented in the WFU PickAtlas tool [34], [35].

### Logistic regression analyses

**Figure 2:**
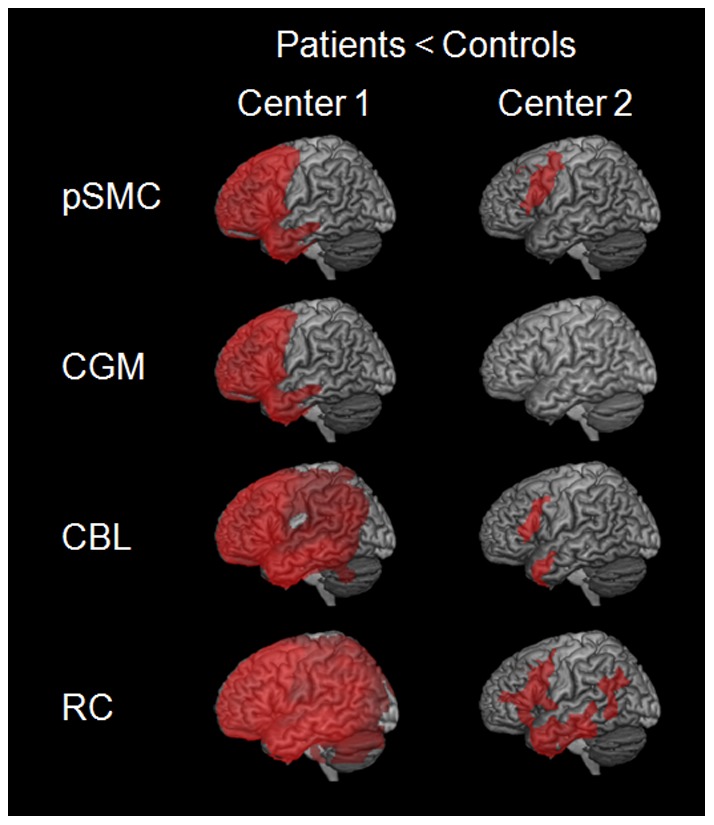
Regions showing a significant decrease in glucose metabolism in frontotemporal lobar degeneration compared to control subjects in center 1 (left) and 2 (right) after intensity normalization to different reference regions, in particular primary sensorimotor cortex (SMC), cerebral global mean (CGM), cerebellum (CBL), and reference cluster (RC).

Accuracy for discrimination between FTLD patients and control subjects was calculated using logistic regressions with split-half cross-validation separately for each center and normalization. Thereby, the diagnostic labels were used as the dependent variable. As independent variable we used individual mean FDG-uptake values extracted after each type of intensity normalization. All mean values were extracted from the hypometabolic pattern detected after CBL normalization (Figure 2) as a kind of gold standard. Indeed, this reference region has been widely applied in FDG-PET imaging of FTLD and has been shown to be the most sensitive approach for diagnostic purposes [2]. Additionally, we extracted mean values from an overlap of hypometabolic patterns detected using all reference regions at center 1. This pattern included bilateral frontal and anterior temporal regions. To obtain accuracy distributions, the split-half cross-validation procedure was repeated 5000 times by randomly assigning patients and control subjects to training and testing datasets and calculating prediction accuracies for the data not used for training. Accuracy, sensitivity and specificity distributions obtained for FTLD patients and control subjects after normalization to each reference region were compared to each other using t-tests for independent samples and applying a significance threshold of p<.05 (Bonferroni corrected for multiple comparisons). Logistic regressions and t-tests comparing the obtained accuracies were implemented using functions provided by Matlab.

### Demographic characteristics

Group comparisons for age were performed by conducting Student's t-tests with a two-sided significance threshold of p<0.05 implemented in Matlab 7.11. Group differences regarding gender were evaluated using a chi-square test for independence using the commercial software package SPSS 17.0 (http://www.spss.com/statistics/).

## Results

### Demographic results

The chi-square test for independence did not reveal any statistical differences in gender between the groups at center 2 [χ2(1) = .24; p = 0.628]. Gender distribution between FTLD patients and control subjects differed significantly at center 1 [χ2(21) = 7.82; p = 0.005]. There was a minor but significant difference in age between control subjects and FTLD patients at center 2 [t(22) = −3.19; p = 0.004], which was taken generally into account in the following analyses by including age as a covariate. No significant difference in age was observed at center 1 [t(81) = 0.98; p = 0.328].

### Voxel-based analyses

The comparison of FTLD patients and control subjects at center 1 revealed a significantly lower relative FDG uptake in bilateral frontal, anterior temporal, cingulate cortices, caudate nucleus and thalamus for CGM and SMC (Figure 2 and Table 2). Decreased relative FDG-uptake was more pronounced, both in respect to extent and height, after intensity normalization to CBL and RC, sparring only occipital, cerebellar and vermis regions after normalization to CBL while only the vermis after normalization to RC. For data from center 2, effects were generally smaller. Left-hemispheric lower relative tracer uptake was detected in FTLD patients in inferior and middle frontal gyrus, pars triangularis, anterior superior, middle, inferior temporal gyrus and precentral gyrus after normalization to CBL. Normalization to RC additionally revealed lower relative FDG uptake in left fusiform gyrus, supramarginal gyrus, frontal inferior operculum, angular gyrus, inferior orbital gyrus, insula, rolandic operculum, parahippocampal and inferior parietal regions. Normalization to SMC revealed lower relative tracer uptake only in left middle and superior frontal gyrus, parts of the precentral gyrus, pars triangularis and operculum. No significant cluster was detected in patients from center 2 after normalization to CGM.

**Table 2:** Hypometabolism in patients with frontotemporal degeneration after intensity normalization to different reference regions.

	Center 1		Center 2	
	Cluster extent	Peak t-value	Cluster extent	Peak t-value
CGM	36806	8.7	n.s.	n.s.
SMC	46215	8.9	1652	5.0
Cerebellum	108383	9.5	1281	4.3
RC	167801	10.9	7216	5.1

The cluster extent is represented by the sum of all clusters (in voxels) which exceeded an threshold p<0.001 (uncorrected) at voxel level and p<0.05 (family-wise error corrected) at cluster level. CGM cerebral global mean, SMC primary sensorimotor cortex, RC reference cluster.

n.s. not significant.

**Figure 3:**
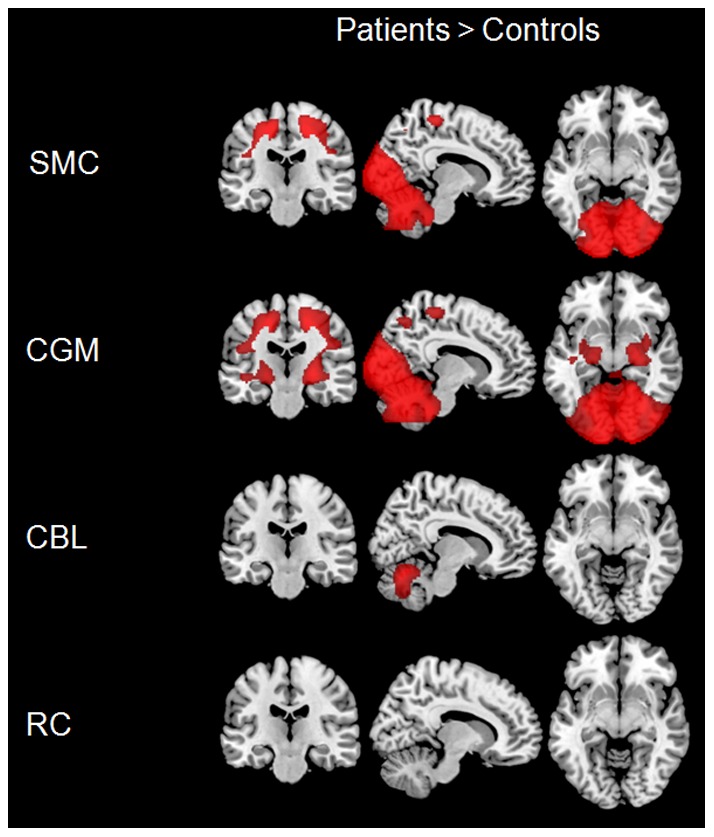
Regions showing an increase in glucose metabolism in frontotemporal lobar degeneration compared to control subjects in center 1 after intensity normalization to different reference regions, in particular primary sensorimotor cortex (SMC), cerebral global mean (CGM), cerebellum (CBL), and reference cluster (RC).

For data from center 1, the opposite contrast investigating increases in glucose uptake in patients resulted in appearance of significantly higher FDG uptake in CBL, primary sensorimotor, occipital and parietal regions of patients after normalization to SMC and CGM (Figure 3). After normalization to CBL higher FDG uptake was only detected in vermis and parts of CBL. No significant clusters were observed after normalization to RC. For data from center 2, no significant clusters were observed in FTLD patients after any type of normalization.

### Logistic regression

**Figure 4:**
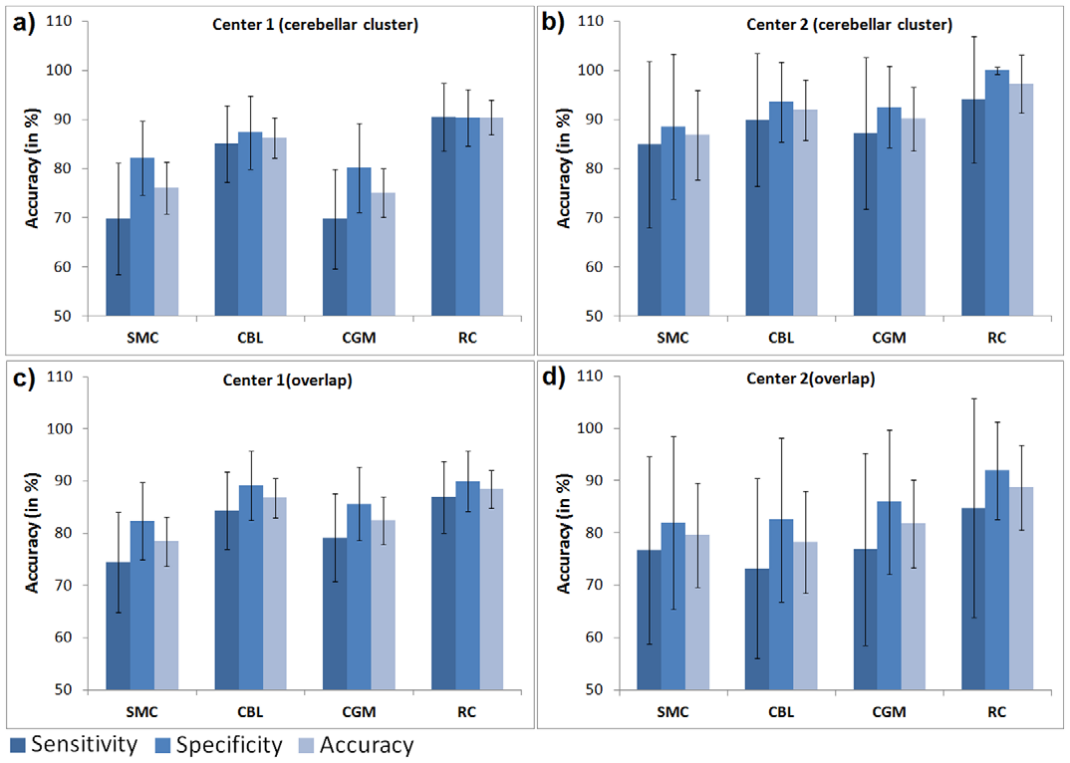
Accuracies, sensitivities and specificities are displayed for each type of intensity normalization. Accuracies, sensitivities and specificities were obtained using mean uptake values extracted from the cerebellar cluster (center 1 (a), center 2 (b)) and from the overlap of all clusters detected at center 1 (center 1 (c), center 2 (d)) for differentiation between frontotemporal lobar degeneration patients and control subjects using logistic regressions. Mean values and standard deviations (error bars) obtained after 5000 permutations using split-half cross-validation are displayed. SMC primary sensorimotor cortex, CBL cerebellum, CGM cerebral global mean, RC reference cluster.

In analyses with mean uptake values extracted from the hypometabolic pattern detected using CBL as a reference region normalization to RC provided significantly higher (p<0.001) mean accuracies for discrimination between FTLD patients and control subjects in both cohorts (center 1: 90.4%; center 2: 97.3%) as compared to all other reference regions (Figure 4). Normalization to CBL performed second best in both centers (center 1: 86.2%; center 2: 91.9%). CGM (90.1%) was superior to SMC (86.9%) at center 2 whilst the opposite was the case at center 1 (CGM: 75%; SMC: 76.1%). The differences between all normalization procedures were highly significant (p<0.001). When comparing sensitivities and specificities between different normalization procedures, again all comparisons with the exception of the comparison of sensitivities for CGM and SMC normalizations at center 1 (p = 1.0) were highly significant (p<.001) showing the same order as the total accuracies. At both centers, sensitivities were significantly lower (p<0.001) than specificities after normalization to all reference regions with the exception of normalization to RC at center 1 (p = 1.0).

Logistic regressions calculated in center 1 and center 2 based on the overlap of all clusters detected in the corresponding center revealed highly similar accuracy distributions (center 1: RC: 88.5%; CBL: 86.8%; CGM: 82.4%; SMC: 78.4%; center 2: RC: 88.7%; CBL: 78.3%; CGM: 81.8%; SMC: 79.6%) with the only difference that CGM was now superior to SMC at center 2 (Figure 4). All differences were again highly significant (p<0.001). Similarly, the obtained sensitivities and specificities differed significantly (p<.001) between all normalization procedures except for the comparison of sensitivities obtained after CGM and SMC normalization (p = 1.0) and of specificities for the comparison of CBL and SMC normalization (p = 1.0), both at center 2. At both centers, all sensitivities were significantly lower than the specificities (p<.001).

## Discussion

In the present study, we compared performance of data-driven RC normalization [6] with that of normalization to CGM, CBL and SMC in mild FTLD. The major finding is that RC normalization allowed for a more accurate detection of early FTLD related metabolic deficits than normalization to any common reference region. Furthermore, this superior performance held its standing after cross-validation, i.e. when a representative RC cluster was applied to image data from another center that were acquired with a different protocol.

The proposed method of intensity normalization is in essence based on the assumption that CGM metabolism is significantly decreased in patients with neurodegenerative disorders. Although, to the best of our knowledge, this has not been explicitly reported in FTLD, this is rather expected, taking into account the presence of rather extensive regional metabolic deficits already at mild disease stages [20], [36]. When comparing patients vs. healthy subjects, intensity normalization to CGM may lead to two unwanted effects in the patient group: underestimation of true regional hypometabolism or -perfusion and detection of apparent (but false) regional hypermetabolism or -perfusion due solely to overcorrection of global variation [37], [38]. Here we show that both effects, in an extensive manner, indeed take place in FTLD, even at a mild disease stage. Moreover, under the same probability threshold, the extent of apparent hypermetabolism is substantially larger than that of true hypometabolism (data not shown). This observation suggests that so-called global normalization alone should not be applied in the comparison of FTLD patients vs. control subjects, even at mild disease stages. However, this procedure may be beneficial in detecting regions that are relatively preserved in a given neurodegenerative disease [6], [9], [27]. Here we demonstrate that intensity scaling to such regions increases statistical power in detecting FTLD-related regional deficits. Moreover, this gain appears substantial enough to significantly increase accuracy of discrimination between patients with mild FTLD and healthy elderly subjects.

While CGM and CBL have been most widely used for scaling of tracer uptake in FTLD (e.g., CGM: [2], [19], [39], [40]; cerebellum: [2], [17], [41], [42], the SMC was included, because it was shown to be a valuable reference area in AD [1]. As expected, CBL was superior to CGM, but it was also superior to SMC. This finding is in line with a quantitative FDG-PET study on FTLD [4] as well as with evidence from histopathological literature [43], [44]. The fact that our RC was also localized within the CBL further supports the view that CBL is a reference area of choice in FTLD. Our data indicate, however, that parts of the cerebellar cortex are probably also hypometabolic. Thus, the RC identifies the cerebellar subregion that is most conserved, and therefore provides the least biased normalization. This interpretation also explains why the RC performs better than employing an a priori CBL region in its entirety. In particular, we found that FDG uptake in the anterior-medial parts of the cerebellar hemispheres, as well as in the vermis, was most preserved in our patients with mild FTLD. Such a pattern might be a result of cerebellar diaschisis with a specific degeneration of cerebropontine-cerebellar pathways [45]. It should be noted, however, that FTLD is a heterogeneous group of diseases, so there might be subtype-specific patterns of preserved glucose metabolism.

In a second step, we asked if the derived RC is representative enough to improve detection of hypometabolism in an independent cohort from another center. Indeed, RC normalization was still significantly superior to other commonly used reference regions. Of note, the image data from the independent cohort were acquired using different acquisition protocols. Furthermore, as compared to control subjects at center 1, the control group at center 2 consisted of individuals with subjective cognitive complaints. Indeed, the latter situation is even closer to routine clinical practice, where a differentiation between such subjects and patients with a suspected neurodegenerative disorder might be really challenging. Nonetheless, RC normalization significantly improved discrimination between these control subjects and patients with mild FTLD. These findings suggest that the derived FTLD-specific RC is rather robust to between-center variability in data acquisition and clinical assessments. Thus, the cluster can be used in independent centers, both on a group and single subject basis.

This study has some limitations. Firstly, the sample size from the center 2 was relatively small. However, given the observation of a similar superiority of the RC approach in a second substantially bigger sample we feel confident that the results reflect a true difference between normalization procedures. However, the substantially smaller sample size in center 2 might explain the lower degree of hypometabolism detected in this FTLD cohort. Secondly, according to standards of clinical work, our PET data were acquired without arterial or venous blood sampling. As we analyzed only relative FDG-uptake, we cannot exclude the presence of true hypermetabolism in our patients with FTLD. To the best of our knowledge, however, there have been no reports describing increases in resting state regional metabolism in subjects with FTLD. Nonetheless, caution is needed when applying the RC approach in psychiatric or neurological disorders where hypermetabolism or hyperperfusion might be part of the disease process. Furthermore, accuracy values provided by RC normalization at center 1 might be overoptimistic, as the RC was obtained on the basis of the same image data. Yet, application of the same cluster to an independent cohort of subjects from center 2 resulted in a similar accuracy increase. Of note, this was also the case when normalizing image data from center 1 to a RC obtained at center 2 (data not shown). Although there was a substantial overlap between two clusters, we chose the one from center 1 to be applied in major analyses, as it is based on substantially more cases. Furthermore, we did not apply correction for partial volume effects. However, in our previous work on the same study sample from center 2 we have shown that partial volume effect correction has only little effect on the comparison of different normalization procedures [2]. Finally, and perhaps most importantly, the methodology presented in this paper is not generalized to the clinical practice in which an expert in nuclear medicine encounters with a large spectrum of conditions/disorders on a single-subject basis. This essential issue should be addressed in future studies with a different design. While we fully admit the indispensable value of visual scan reading in clinical settings, an increasing role of automated image-based classification procedures that can be applied to analyses of large-scale image databases in a user-independent manner should be recognized and noted [46], [47]. Logistic regression analyses such as those applied in the present study are a common step in such classification algorithms. However, it is important to note that the purpose of our study was not to develop or to establish a classification algorithm but rather to the examine how classification accuracy of automated classification procedures on the same data is influenced by the choice of reference region for intensity normalization.

In summary, we found that data-driven RC normalization improves detection of glucose hypometabolism in mild FTLD. Such an improvement appears substantial enough to increase accuracy of discrimination between FTLD patients and healthy subjects, also in an independent cohort. The established FTLD-specific cluster can be used for intensity normalization at independent imaging centers. The cluster as binary image is available for free download at http://www.unil.ch/webdav/site/lren/shared/Juergen/wRefClus_Munich_41Pat42Kon_FWE0.000001.nii.
